# Effect of green tea on blood glucose levels and serum proteomic patterns in diabetic (db/db) mice and on glucose metabolism in healthy humans

**DOI:** 10.1186/1471-2210-4-18

**Published:** 2004-08-26

**Authors:** Hiroshi Tsuneki, Mitsuyo Ishizuka, Miki Terasawa, Jin-Bin Wu, Toshiyasu Sasaoka, Ikuko Kimura

**Affiliations:** 1 Department of Clinical Pharmacology, Toyama Medical and Pharmaceutical University, Toyama 930-0194, Japan; 2 Toyama College, Toyama 930-0193, Japan; 3 China Medical College, Taichung, Taiwan Republic of China

## Abstract

**Background:**

Green tea is widely consumed in Asian countries and is becoming increasingly popular in Western countries. Epidemiologically, it has been suggested that green tea consumption prevents type 2 diabetes. The present study was aimed at providing evidence of improvement in glucose metabolism in diabetic mice and healthy humans upon green tea consumption.

**Results:**

Green tea promoted glucose metabolism in healthy human volunteers at 1.5 g/body in oral glucose tolerance tests. Green tea also lowered blood glucose levels in diabetic db+/db+ mice and streptozotocin-diabetic mice 2–6 h after administration at 300 mg/kg without affecting serum insulin level, whereas no effect was observed in control mice (+m/+m and normal ddY mice). The serum protein profiles of db+/db+ and +m/+m mice were analyzed for the first time by SELDI (surface-enhanced laser desorption/ionization)-TOF (time-of-flight)-MS (mass spectrometry), and then compared to investigate any effects of oral green tea administration on serum proteins. The protein profiles in db+/db+ mice showed that the spectral peak intensities at the mass/charge ratios (m/z) of 4119, 4203, 4206, 4211, 4579, 9311 and 18691 were >3 times lower, and those of 13075, 17406, 17407, 17418, 17622, 18431 and 26100 were >3 times higher than respective peak intensities in +m/+m mice. When green tea was administered to db+/db+ mice, the peak intensities were markedly decreased at m/z 11651 and 11863, and slightly decreased at m/z 4212. The peak intensities at 7495, 7595, 7808, 14983, 15614, 31204 were markedly increased after the administration.

**Conclusion:**

The present study provides evidence that green tea has an antidiabetic effect. Although we could not find simple reversed effect of green tea on the diabetes-induced modifications of the levels of several serum proteins, we found that the 4211 (4212) Da protein level that was decreased in the diabetic state was further decreased after green tea administration. This is the first report demonstrating that a certain serum protein may be involved in the antihyperglycemic effect of green tea. The contribution of this protein should be further studied.

## Background

Green tea (leaves of *Camellia sinensis*, Theaceae) is a popular beverage in East Asia, and also used as a herbal remedy in Europe and North America. Green tea is considered to be antiinflammatory, antioxidative, antimutagenic, and anticarcinogenic [1,2], and can prevent cardiac disorders. Epidemiologically, it has been suggested that green tea consumption prevents type 2 diabetes. The amelioration of insulin resistance by green tea is associated with the increased expression level of glucose transporter IV in a fructose-fed rat [3]. Green tea extract contains polyphenols (e.g., catechin, epicatechin, epigallocatechin, and their gallates), teanin and caffeine. The extract also includes pyrroloquinoline quinone, a newly discovered vitamin [4]. Some constituent components have been shown to enhance the basal and insulin-stimulated glucose uptake of rat adipocytes [5], to inhibit intestinal glucose uptake by inhibiting the sodium-dependent glucose transporter of rabbit intestinal epithelial cells [6], and to reduce serum glucose level in alloxan-diabetic rats [7]. Controversially, caffeine acutely lowers insulin sensitivity in humans [8].

The present study was aimed at providing evidence of the improvement in glucose metabolism in humans and diabetic mice upon green tea consumption. Furthermore, to investigate whether some serum proteins mediate the effects of green tea on hyperglycemia in the diabetic state, proteomic analyses were performed using SELDI (surface enhanced laser desorption/ionization)-TOF (time-of-flight)-MS (mass spectrometry), because the SELDI-TOF-MS enables the measurement of the relative abundance of proteins of various sizes in a blood sample. Here, we compared the proteomic patterns of sera from diabetic db+/db+ and wild-type mice, and further investigated the influence of green tea administration on the proteomic patterns of serum in diabetic mice. If the diabetic modifications of serum protein levels are reversed by green tea administration, these affected proteins can be used as therapeutic targets in diabetes.

## Results

### Oral glucose tolerance test (OGTT)

**Figure 1:**
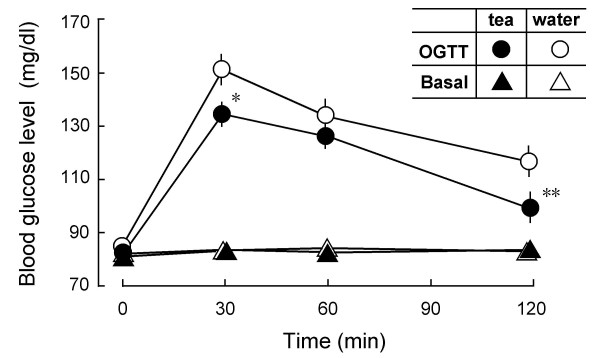
**Increase in glucose metabolism in healthy humans administered with green tea. **Oral glucose tolerance curves (OGTT, n = 22) and basal blood glucose levels (Basal, n = 25) before and 30, 60 and 120 min after administration with either a suspension of green tea powder (●) or hot water (○). All participants were fasted since the last supper before starting the experiment at 9:30 a.m. In the glucose tolerance test, 225 ml of Trelan-G75 containing 75 g glucose was perorally administered 10 min after drinking a suspension of green tea powder (1.5 g/150 ml hot water) or hot water (150 ml). Effect of green tea or hot water on basal blood glucose levels was examined without administrating Trelan-G75. Blood glucose levels (mg/dl) measured were averaged in each treatment group, and compared by cross-over test. Values represent the means ± S.E.M. *P < 0.05, **P < 0.01; significantly different from the vehicle control at each time-point, determined by one-way ANOVA, followed by Scheffé's test.

We investigated whether oral glucose tolerance is improved in healthy volunteers by drinking a suspension of green tea powder. Blood glucose levels were measured before and 30, 60, and 120 min after drinking 1.5 g of green tea. The effects of hot water without tea powder on blood glucose level in the same participants were also investigated. Fig. 1 shows that glucose tolerance was substantially improved with tea administration compared with hot water administration. In detail, glucose metabolism increased in 14 participants, remained unchanged in 3 participants, and worsened in 5 participants. Basal blood glucose levels (BGLs) did not significantly change in all participants (Fig. 1).

### Antihyperglycemic effects of green tea in diabetic mice

**Figure 2:**
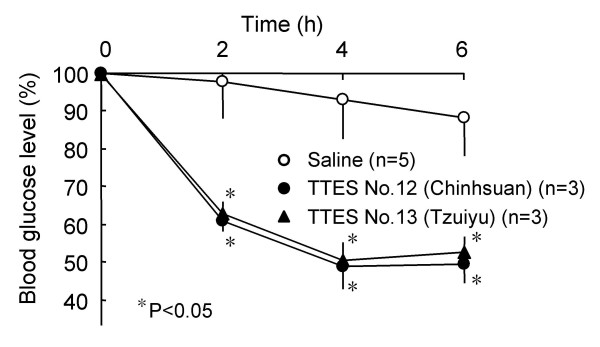
**Antihyperglycemic effects of green tea leaf extracts in the fasting STZ-diabetic ddY mice. **The dried extracts of fresh tea leaves prepared at the Taiwan Tea Experiment Station [TTES, Cultivar No. 12 (Chinhsuan) and No.13 (Tzuiyu)] were dissolved in saline and perorally administered at 300 mg/kg into STZ-diabetic ddY mice in the fasting state. Blood glucose levels 2 h, 4 h, and 6 h after administration of green tea leaf extracts are expressed as percentages of the glucose levels at 0 h (before the administration) in each mouse, and then averaged. Values represent the means ± S.E.M. (n = 3–5). *P < 0.05; significantly different from the blood glucose levels of STZ-diabetic mice administered with saline (control) at each time-point, determined by one-way ANOVA, followed by Scheffé's test.

The crude extract of fresh tea leaves picked from either No.12 or No. 13 cultivar at 300 mg/kg significantly lowered BGL 2–6 h after peroral administration in streptozotocin (STZ)-diabetic ddY mice in the fasting state (Fig. 2). In the saline-treated control group (n = 5), BGL slightly changed from 273 ± 28 mg/dl to 241 ± 28 mg/dl after 6 h, whereas in the green-tea (No. 12)-treated group (n = 3), BGL markedly changed from 235 ± 15 mg/dl to 116 ± 12 mg/dl. Similarly, BGL markedly changed from 201 ± 10 mg/dl to106 ± 8 mg/dl after 6 h in the green-tea (No. 13)-treated group (n = 3).

**Figure 3:**
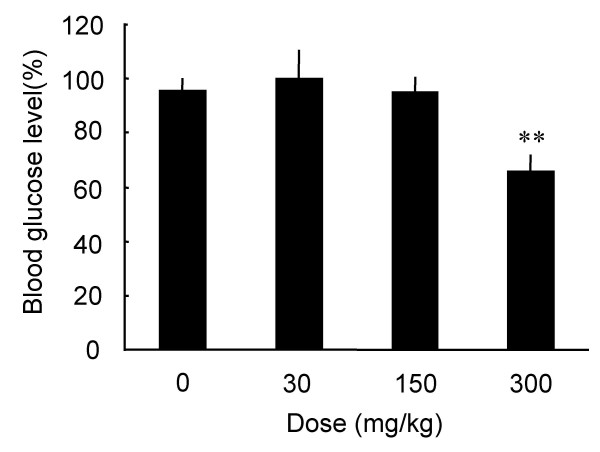
**Antihyperglycemic effects of green tea powder suspension in the fasting STZ-diabetic ddY mice. **STZ-diabetic ddY mice were perorally administered with a suspension of green tea powder at 30, 150, and 300 mg/kg in the fasting state. Blood glucose levels 2 h after the administration of green tea are expressed as percentages of the glucose level 0 h before the administration in each mouse, and then averaged. Values represent the means ± S.E.M. (n = 6 – 20). **P < 0.01; significantly different from the blood glucose levels of STZ-diabetic mice administered with saline (control, 0 mg/kg of green tea), by one-way ANOVA, followed by Scheffé's test.

**Figure 4:**
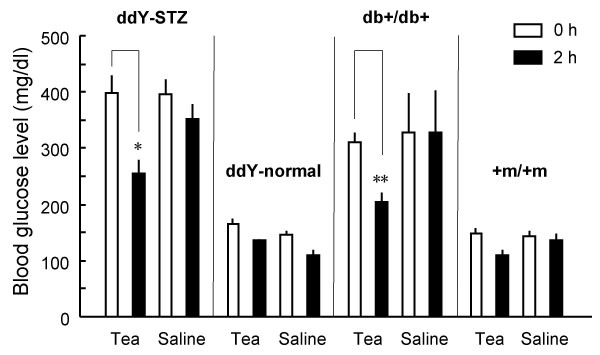
**Blood-glucose-lowering effects of green tea in diabetic mice but not in normal mice. **STZ-diabetic ddY, normal ddY, diabetic db+/db+, and wild-type (+m/+m) mice were perorally administered with either a suspension of green tea powder at 300 mg/kg or saline in the fasting state. Blood glucose levels (mg/dl) were measured before and 2 h after the administration, and then averaged. Values represent the means ± S.E.M. (n = 5 – 21). *P < 0.05, **P < 0.01; significantly different from values at 0 h (before the administration), by one-way ANOVA, followed by Scheffé's test.

The antihyperglycemic effects of green tea powder suspension at 30, 150, and 300 mg/kg were examined 2 h after administration to the STZ-diabetic mice in the fasting state. As shown in Fig. 3, green tea tended to lower BGL at 150 mg/kg, and significantly lowered BGL at 300 mg/kg. Furthermore, the effect of green tea was compared among different groups of mice, i.e., STZ-diabetic ddY mice, normal ddY mice, diabetic db+/db+ mice and +m/+m mice (Fig. 4). BGLs were significantly lowered in both STZ-diabetic mice and db+/db+ mice 2 h after administration of green tea powder suspension (300 mg/kg), whereas no significant changes were observed in normal ddY mice and +m/+m mice.

No elevation of blood insulin levels was observed during the decrease in BGL either by tea leaf extract or by green tea suspension in STZ-diabetic mice (data not shown). These results suggest that both fresh green tea leaf extract and green tea suspension lower BGL without changing serum insulin concentration.

### Serum protein profiles altered in diabetic db+/db+ mice

**Figure 5:**
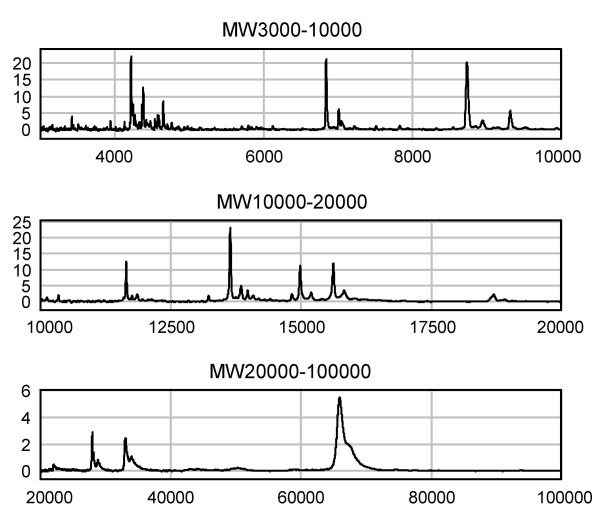
**Analysis of proteomic profiles in mouse serum using SELDI-TOF-MS. **Serum samples from fasting wild-type (+m/+m) mice were loaded onto the spots of CM10 (a cationic exchanger, pH4) ProteinChip arrays. The spots were analyzed using the SELDI ProteinChip system on three different ranges: m/z 3000 – 10000, m/z 10000 – 20000 and m/z 20000 – 100000. The y-axis of the spectra indicates the mass-to-charge ratio (m/z) of protonated proteins, and the x-axis indicates the relative intensities of mass spectral signals. Note: The intensity of each peak is directly proportional to the amount of protein, but the peak intensities could not be comparable among the different proteins because of the difference in ionization.

**Figure 6:**
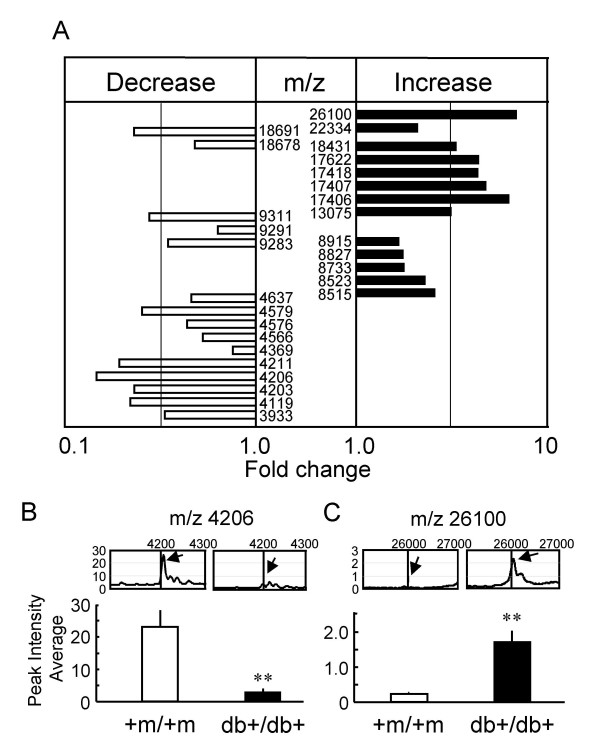
**Proteomic analyses demonstrating the differences in protein profiles of sera from diabetic and wild-type mice. **Serum samples from fasting diabetic (db+/db+) mice and fasting wild-type (+m/+m) mice were loaded onto ProteinChip arrays. **(A) **List of peaks of proteins and/or peptides with indicated m/z values, the peak intensities of which were significantly changed in the diabetic state. Relative peak intensities were averaged (n = 8). The fold changes are presented as ratios of the peak intensities at indicated m/z values in db+/db+ to those in the +m/+m mice. Peaks at m/z 4203, 4576, 8515, 9291, 17406 and 18678 were obtained using CM10 ProteinChip (pH4). Peaks at 8733 and 9311 m/z were obtained using CM10 ProteinChip (pH7). Peaks at m/z 3933, 4119, 4206, 4369, 4566, 4579, 4637, 8523, 8827, 8915, 9283, 13075, 17407, 17418, 17622, 18431, 18691, 22334 and 26100 were obtained using Q10 ProteinChip. Peak at m/z 4211 was obtained using IMAC30. The chips were analyzed by SELDI-TOF-MS. **(B) **and **(C) **Typical data of relative peak intensities in +m/+m and db+/db+ mouse sera (upper, representative of 4–8 independent observations) and the peak intensity averages at m/z 4206 and 26100 (lower, n = 4 for +m/+m, n = 8 for db+/db+). The analyzed peak is indicated by arrows in the data of mass spectral signals. **P < 0.01; significantly different from the peak in wild-type mice, by unpaired *t*-test.

To explore the mechanisms underlying the antihyperglycemic effect of green tea, proteomic patterns of sera from diabetic (db+/db+) and wild-type (+m/+m) mice were first investigated and compared to identify peaks specific to the diabetic state using SELDI-TOF-MS. Samples of sera from fasting +m/+m mice were loaded onto CM10 (a cationic exchanger, pH4) ProteinChip arrays (Fig. 5). The other types of ProteinChip Array, i.e., CM10 (a cationic exchanger, pH7), Q10 (an anionic exchanger, pH8) and IMAC30-Cu^2+ ^(immobilized metal affinity chromatography, pH7), were also used to fractionate proteins in the serum. Then, these arrays were analyzed using a ProteinChip system, and the amount of individual serum proteins was estimated from the peak intensity of the mass spectral signal and the mass/charge ratio (m/z) equivalent to the molecular weight of each protein. All the spectral peaks of serum proteins from diabetic mice, which were significantly different (P < 0.05, by unpaired *t*-test) in intensities from those of serum proteins from wild-type mice, are shown in Fig. 6A: the peak intensities of proteins at m/z 4119, 4203, 4206, 4211, 4579, 9311 and 18691 were >3 times lower, and those at m/z 13075, 17406, 17407, 17418, 17622, 18431, 26100 were >3 times higher than respective peak intensities of proteins of sera from +m/+m mice. Typical spectra are shown in Figs. 6B and 6C (see additional file 1 concerning the other peaks).

### Modification of serum protein profiles of diabetic db+/db+ mice by green tea

**Figure 7:**
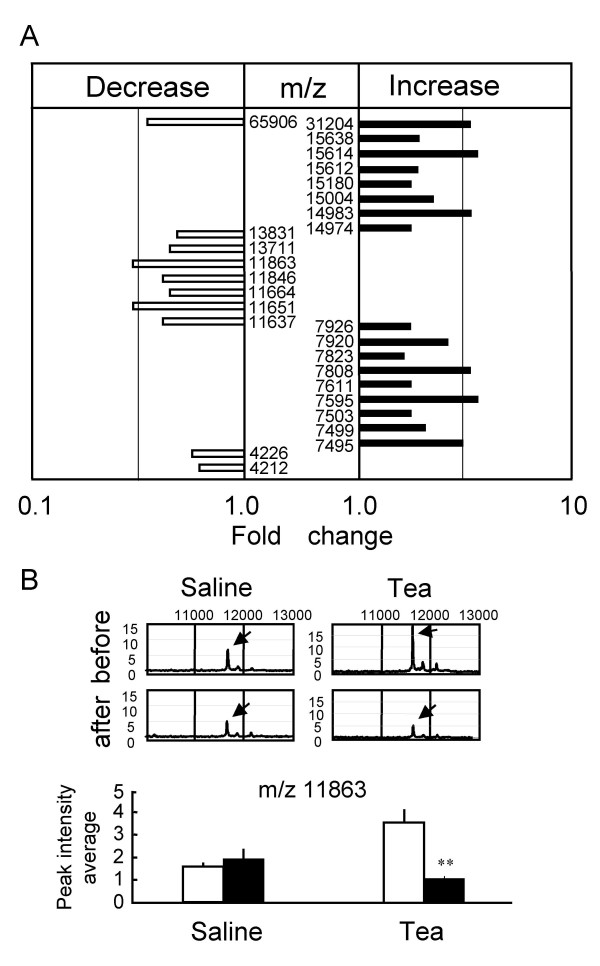
**Changes in serum protein profiles in db+/db+ mice by administration of green tea. **Serum samples from fasting diabetic (db+/db+) mice 2 h after administration of green tea suspension were loaded onto ProteinChip Arrays. The chips were analyzed using SELDI-TOF-MS. **(A) **List of peaks of proteins and/or peptides with indicated m/z values, the peak intensities of which were significantly changed by the green tea administration. Relative peak intensities were averaged (n = 4). The fold changes are presented as ratios of the peak intensities at indicated m/z values 2 h after to before green tea administration. Peaks at m/z 7495, 7595, 7808, 7920, 14983, 15612 and 15614 were obtained using CM10 ProteinChip (pH4), whereas those at m/z 7503, 7611, 7823, 7926, 11651, 11664, 11863, 15004 and 15638 were obtained using CM10 ProteinChip (pH7). Peaks at m/z 4212, 4226, 7499, 11637, 11846, 13711, 13831, 14974, 15180, 31204 and 65906 were obtained using IMAC30 ProteinChip. **(B) **Typical data of relative peak intensities (upper, representative of 4 independent observations) and the peak intensity average at m/z 11863 (lower, n = 4) after green tea administration and saline control. The analyzed peak is indicated by arrows in the data of mass spectral signals. **P < 0.01; significantly different from the peak obtained before the administration, by unpaired *t*-test.

**Figure 8:**
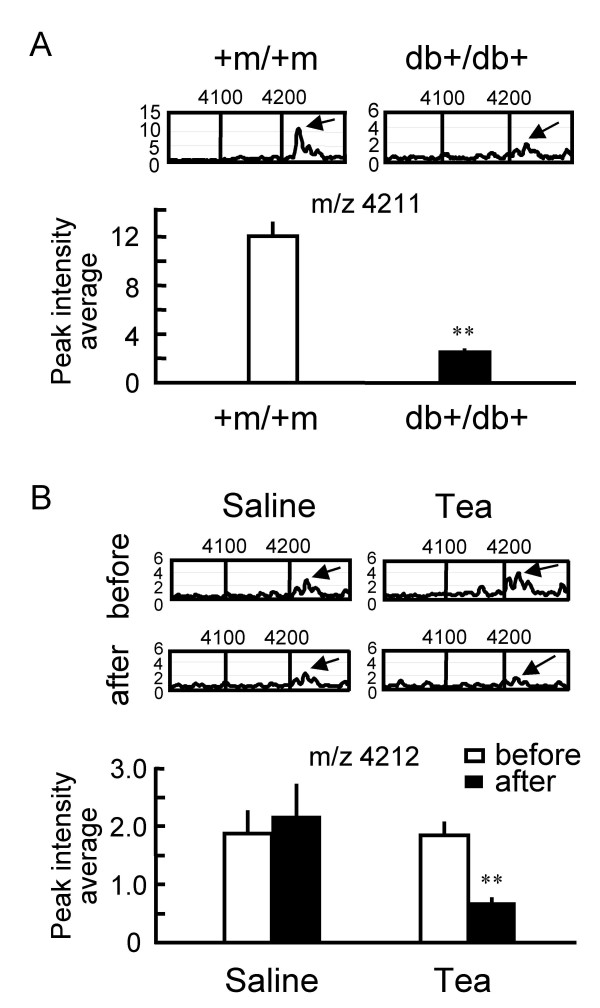
**Changes in peak intensity at m/z 4211 in diabetic state and after green tea administration. (A) **Decrease in the peak intensity at m/z 4211 in serum samples from fasting diabetic (db+/db+) mice, compared with that of the fasting wild-type (+m/+m) mice. Typical data of relative peak intensities in +m/+m and db+/db+ mouse sera (upper, representative of 4–8 independent observations) and the averaged intensities of the peak at m/z 4211 (lower, n = 4 for +m/+m, n = 8 for db+/db+) indicated above by arrows. **P < 0.01; significantly different from the peak in wild-type mice, by unpaired *t*-test. **(B) **Decrease in the peak intensity at m/z 4212 in serum samples from fasting db+/db+ mice 2 h after green tea administration. Typical data of relative peak intensity (upper, representative of 4 independent observations) and the averaged intensities of the peak at m/z 4212 (lower, n = 4) after administration with either green tea or saline. **P < 0.01; significantly different from the peak obtained before the administration, by unpaired *t*-test.

Serum protein profiles of db+/db+ mice were investigated 2 h after green tea administration, and compared with those administered with saline (control). Again, we confirmed a significant decrease in BGL by green tea in the blood samples used in these proteomic analyses (data not shown). All the protein signals that were significantly changed (P < 0.05, by unpaired *t*-test) in terms of peak intensity by the green tea administration, but not by the saline administration, are described in Fig. 7A. The serum proteomic patterns of the green-tea-treated group demonstrate that the peak intensities of proteins at m/z 11651 and 11863 decreased to less than one third after 2 h of tea administration, whereas no significant changes were observed in the control group. Typical spectra are shown in Fig. 7B (see additional file 2 for the change at m/z 11651). When the results in Fig. 7A are compared with those in Fig. 6A to identify protein peaks specific for diabetes and which are sensitive to green tea administration, a peak at m/z 4211 (4212) was found (Fig. 8). The intensity of this peak was significantly lowered in the diabetic state (Fig. 8A), and was significantly decreased 2 h after green tea administration (Fig. 8B).

**Figure 9:**
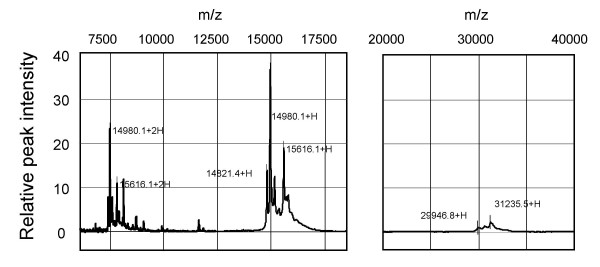
**Hemoglobin-related multiple SELDI-TOF-MS signals in db+/db+ mice. **Samples of sera from nontreated db+/db+ mice in the fasting state were loaded onto ProteinChip arrays. Left panel: hemoglobin-related multi-MS signals. From the average of 8 data, the signals were detected from m/z 14974 to 15638 (+H) using CM10 ProteinChip (pH7). The double-charged m/z values appear to be observed from m/z 7495 to 7823 (+2H). These values may correspond to hemoglobin α- and β-chains, and other hemoglobin-related proteins. Right panel: multi-MS signals observed at approximately double the single-charged m/z values for the hemoglobin-related signals, using IMAC ProteinChip. These signals may correspond to the dimers of hemoglobin α- and β-chains.

The relative peak intensities of hemoglobin-related multisignals ranging from m/z 14974 to 15638 [9] were increased by green tea administration (Fig. 7A). Parallel changes were observed at half and double these m/z values (Fig. 7A, 9) (see additional file 2 concerning these changes). Since multicharged protein ions are apparently observed as proteins of different sizes in the mass spectrometry, these changes seem to reflect the modification of one group of proteins (hemoglobin-related proteins) by green tea intake.

## Discussion

Green tea is widely consumed in Asian countries, while black tea is most popular in Western countries. The manufacturing process of green tea differs from that of black tea because freshly picked young leaves of the tea are immediately steamed. This process destroys the enzymes responsible for breaking down the color pigments in the leaves and allows the tea to maintain its green color during the subsequent rolling and drying processes. The amounts of constituent compounds are slightly different from those of black tea. Pharmacological studies using constituent compounds in green tea have been recently reviewed by Kaszkin et al. [10]. Green tea extracts are more stable than pure epigallocatechin gallate, the major constituents of green tea, because of the presence of other antioxidant constituents in the extract [10]. In general, herbal medicines are complex mixtures of different compounds that often act in a synergistic fashion and exert their full beneficial effect as total extracts [11].

In the present study, we demonstrated that green tea produces an antihyperglycemic effect without affecting insulin secretion in STZ-diabetic mice. We therefore explore the mechanism underlying the green tea effect by investigating the serum protein profiles of db+/db+ mice, a genetic model of type 2 diabetes, using SELDI-TOF-MS. First, we performed a preliminary analysis to determine the peaks (molecular weights) of biomarker proteins that were abnormally contained in the serum of diabetic mice, without identifying individual serum proteins. As a result, we found that the levels of several serum proteins were significantly altered in the diabetic state. Secondly, we investigated which marker proteins are affected by green tea administration. Despite the changes in the levels of several serum proteins after green tea administration, none of the protein peaks specific for diabetes were sensitive to the administration, except for a peak at m/z 4211(4212). The level of this 4211(4212) Da protein was reduced both in the diabetic state and by green tea administration. Thus, no simple reversed effect of green tea on the diabetes-induced modifications of serum protein levels was observed.

The 4211(4212) Da protein has not yet been identified, but only two candidate groups of vertebrate proteins are found by scanning Swiss-Prot database (molecular weight, 4211; molecular weight ranges, 0.1% according to the standard errors of the current MS analysis; pI, 6; pI ranges, 10): pancreatic polypeptide (PP) (primary accession numbers P13083, P37999, P41519, P38000, P11967) and antibacterial peptides [β-defensin C7 precursor (018815), 4 kDa defensin (P56686), cryptidine-5 precursor (P28312), antibacterial peptide BMAP-34 precursor (P56425)]. PP is primarily expressed in the endocrine cells of the pancreas, and the plasma PP concentrations are elevated by food intake [12]. Peripheral PP administration results in a reduction in food intake and an increase in energy expenditure [12]. Interestingly, a previous study revealed that PP-containing cell populations in the islets of Langerhans are reduced in db+/db+ mice with mild hyperinsulinemic diabetes [13], which is consistent with the observed reduction in the intensity of the peak at m/z 4211(4212) in the diabetic state. We speculate that the reduction of BGL by green tea causes a decrease in serum PP concentrations as a means of maintaining energy homeostasis, although more precise studies are required.

The changes in serum protein profiles by green tea also demonstrate the increase in the peak intensities of hemoglobin-related multi-MS signals, suggesting the adverse side effects of green tea, although blood samples from db+/db+ mice tended to exhibit a hemolytic feature compared with those from wild-type mice (data not shown). Interestingly, the hemoglobin-related multi-MS signals shown in Fig. 9 may include hemoglobin α-chains and β-chains [9], their dimers, and possibly variously glycated hemoglobins. Hemoglobin A1c is used as a marker of diabetes in clinical diagnostic tests. Until now, however, the extent of glycation per hemoglobin that practically occurred is not clear. More detailed analyses of serum protein profiles using SELDI-TOF-MS will provide a more useful clinical index of the diabetic state.

We observed that green tea improved oral glucose tolerance in humans. It is therefore likely that green tea is prophylactic against diabetes and ameliorates diabetic hyperglycemia. Green tea consumption at moderate doses may be associated with a reduced risk of type 2 diabetes in apparently healthy individuals by controlling postprandial hyperglycemia.

## Conclusions

The control of postprandial hyperglycemia by green tea can help reduce the risk of type 2 diabetes. In the present study, we provide evidence showing that green tea promotes glucose metabolism in healthy humans, and produces an antihyperglycemic effect in diabetic mice. In addition, we analyzed the serum protein profiles of db+/db+ and +m/+m mice for the first time using SELDI-TOF-MS, and further investigated its association with any effects of oral green tea administration on serum proteins. Among the several proteins that were significantly lowered in the serum of diabetic mice, the 4211(4212) Da protein was significantly decreased after green tea administration. This is the first report demonstrating that a certain serum protein is involved in the antihyperglycemic effect of green tea. The contribution of this protein, therefore, should be further investigated in a future study.

Moreover, we speculate that the observed effects of green tea on BGL are primarily due to the promotion of insulin action in peripheral tissues, such as skeletal muscles and adipocytes. Indeed, a recent paper showed that green tea supplementation for 12 weeks ameliorates insulin resistance and increases glucose transporter IV content in a fructose-fed rat model resembling the human type 2 diabetes mellitus [3]. Since the administration of green tea produced an acute antihyperglycemic effect on BGL in diabetic mice in the present study, additional mechanisms, such as changes in amelioration or enhancement of insulin action, should be clarified in a future study. To elucidate whether new protein synthesis is required for green tea action, it would be useful to examine the influence of protein synthesis inhibitors on the acute antihyperglycemic effect of green tea.

## Methods

### Housing and care of animals

C57BLKS/J db+/db+ mice (male, 9 – 11 weeks old, 36.2 – 46.3 g, BGL: 243–411 mg/dl) and its age-matched control C57BLKS/J +m/+m mice (male, 20.6–23.9 g, BGL: 110–185 mg/dl) were purchased from SLC (Shizuoka, Japan) and used as the type 2 diabetic mouse model. Male ddY mice (4 weeks old, purchased from SLC, Shizuoka, Japan) were singly injected with STZ (150 mg/kg, i.v.), and then used 4–6 weeks after the injection (28.8–38.5 g, BGL: 247–600 mg/dl). The age-matched normal ddY mice (34.7–38.6 g, BGL: 129–197 mg/dl) were also used. The animals were housed (3–5 per cage) under a daily cycle of 12 h light and 12 h darkness, with free access to food and water. Animals were treated as approved by the Toyama Medical and Pharmaceutical University Animal Research Committee, and according to the guidelines for animal experiments established by the Japanese Pharmacological Society.

### Sampling of blood

Mice were deprived of food for 11–14 h. Blood samples (20 μl) were collected from mouse tail veins under ether anesthesia. In experiments for proteomic analyses and serum insulin measurements, blood samples (100 μl) were collected from the orbital venous plexus of mice under ether anesthesia. The food deprivation was continued throughout the measurement of blood glucose levels (until 6 h after the administration with green tea). In the oral glucose tolerance test as described below, blood samples (0.3 μl) were obtained from human skin microvessels using FreeStyle (Nipro, Osaka, Japan).

### Oral glucose tolerance test (OGTT)

Healthy human volunteers (18–24 years old) were fasted 12 h before the starting point of experiments. The participants were perorally administered with either a suspension of green tea powder or hot water at 9:20 a.m. Ten min after the administration (at 9:30 a.m.), the participants were perorally administered with 225 ml of Trelan-G75 (Shimizu Pharmaceuticals Co., Shimizu, Shizuoka) containing 75 g glucose. BGLs were measured before and 30, 60, and 120 min after the administration with Trelan-G75 (glucose). All the subjects enrolled in this study were ethnic Japanese. Before participation, the purpose and risks of the study were carefully explained, and written informed consent was obtained from all the participants. The protocol was approved by the Toyama Medical and Pharmaceutical University Ethics Committee regulating human research.

### Measurement of blood glucose levels and serum insulin levels

BGLs were measured using ANTSENSE II (Horiba, Kyoto, Japan) in the mouse study and using FreeStyle (Nipro, Osaka, Japan) in the human study. Serum insulin levels were measured using an insulin-ELISA kit (Morinaga Seikagaku, Tokyo, Japan). To prepare the serum samples, blood samples from mice (100 μl) were kept on ice for 2 h, centrifuged at 16,000 × *g *for 1 min at 4°C, and its supernatant was immediately separated from the pellet.

### SELDI ProteinChip Analysis

Quantitative serum proteomic profiles were measured with SELDI-TOF-MS (Ciphergen Biosystems, Yokohama, Japan). The mouse serum was prepared as described above. Several types of ProteinChip Array, i.e., Q10 (an anionic exchanger, pH8), CM10 (a cationic exchanger, pH4 and pH7) and IMAC30-Cu^2+ ^(immobilized metal affinity chromatography, pH7) (Ciphergen Biosystems), were used to fractionate proteins in serum. For the proteomic analyses with Q10 ProteinChip array, samples were 10-times diluted with denaturation buffer (7 M urea/ 2 M Thiourea/ 4% CHAPS/ 1% dithiothreitol/ 2% ampholine) and incubated on ice for 10 min, and then 10-times more diluted with buffer of 50 mM Tris-HCl (pH8). When CM10 ProteinChip array was used, samples were 10-times diluted with the denaturation buffer and incubated on ice for 10 min, and then 10-times more diluted with buffer of 100 mM NaOAc (pH4) or 50 mM HEPES (pH7). When IMAC30 ProteinChip array was used, samples were 100-times diluted with phophate-buffered saline. Spots on the different arrays were equilibrated with the buffer used for sample dilution, e.g., Tris-HCl buffer for spots on Q10 array, and each sample solution (70 μl) was loaded onto two separate spots on the arrays. After incubation for 20 min with rotation spots were rinsed with water and air-dried completely. The spots were analyzed using the SELDI ProteinChip system (PBS-IIc, Ciphergen Biosystems). In each sample, data from the two spots were averaged (duplicate assay). If a signal/noise ratio was larger than 2, the peak was considered to reflect the amount of a protein. Quantitative nature of the instrument was confirmed as previously described [14]. The m/z value of each protein peak (the molecular weight of each detected protein) was corrected, based on the m/z values of the external standards of peptides or proteins, the molecular weights of which are known. Standard errors in the estimated molecular weights were less than 0.1%. The axis of abscissa in spectra (Figs 6B, 7B, 8 and 9) indicates the mass-to-charge ratio (m/z) of protonated proteins.

### Reagents

Fresh raw leaves of tea (*Camellia sinensis*, Theaceae) (Cultivar No.12, Chinhsuan and No.13, Tzuiyu) were prepared at the Taiwan Tea Experiment Station, picked in May at Nan-Tou County, and immediately used without drying. The leaves were steeped in hot water (95°C) for 30 min and the filtrate was condensed under reduced pressure. The dry weight of the extracts was determined, and the extracts were dissolved in saline for administration into mice. On the other hand, green tea powder was donated by Fukuju-en (Kyoto, Japan). The particle size (median diameter) of the green tea powder was 2.9 μm, according to the measurement using a Centrifugal Particle Size Analyzer (Shimadzu, Kyoto, Japan). In the human study, the tea powder (1.5 g) was added to 150 ml of hot water (80°C) and then whipped with a bamboo whisk. In the mouse study, the tea powder was suspended in saline at room temperature using a sonicator. The contents of caffeine and catechins in these tea samples were determined by HPLC: the samples were injected into an HPLC column (TSK-GEL, ODS-80T_M_, Tosoh, Tokyo, Japan); the eluent was 10 mM phosphate buffer (pH2.6)/MeCN (gradient: 5 to 15%) at a flow rate of 1.3 ml/min. The data are shown in Table 1.

**Table 1:** Contents of catechins and caffeine in green tea samples (grams per 100 g)

	Green tea powder	No. 12^a^	No. 13^b^
catechins			
C	0.1	1.3	0
EC	0.6	1.9	1.0
ECg	0.6	1.6	0.2
EGC	0.3	0.9	5.8
EGCg	5.6	10.6	3.5
total	7.2	16.3	10.5
caffeine	4.3	4.5	1.7

C: (+)-catechin, EC: (-)-epicatechin, ECg: (-)-epicatechin gallate, EGC: (-)-epigallocatechin, EGCg: (-)-epigallocatechin gallate, total: total catechins. ^a,b ^The dried extract of fresh raw leaves of tea (*Camellia sinensis*, Theaceae) prepared at Cultivar No.12 (a, Chinhsuan) and No. 13 (b, Tzuiyu).

### Statistical analysis

The significance of differences between two groups was assessed by Student's *t*-test, and the differences between multiple groups were assessed by one-way analysis of variance (ANOVA) followed by the Scheffé's multiple range test. Values of P less than 0.05 were considered to be significant. Especially, to determine the significance of the time-dependent effects of green tea (Fig. 1 and 2) on the blood glucose levels, repeated measures ANOVA was performed, and then, if the statistical significance was detected by this analysis, further statistical comparison at each measurement time between the groups was conducted by one-way ANOVA, followed by the Scheffé test.

## Authors' Contributions

IK had the original idea for the study, designed and coordinated the experiments, and wrote the manuscript. JW prepared fresh young leaves of green tea and the hot water extract. MT assisted with measurement of blood glucose and insulin level. MI, HT and TS were involved in study design, data analysis, data interpretation and writing of the manuscript. All authors read and approved the final manuscript.

## Supplementary Material

Additional File 1 **Differences in serum protein profiles between diabetic and wild-type mice **Typical data of relative peak intensities in +m/+m and db+/db+ mouse sera (left 2 panels, representative of 4–8 independent observations) and the peak intensity averages at m/z indicated (right panels; +m/+m: open column, n = 4; db+/db+: closed column, n = 8). The analyzed peak is indicated by arrows in the data of mass spectral signals. **P < 0.01; significantly different from the peak in wild-type mice, by unpaired *t*-test. Types of ProteinChip used were described in the Fig. 6 legend. Click here for file

Additional File 2 **Changes in serum protein profiles of db+/db+ mice after green tea administration **MS spectra shows typical changes in the serum protein profiles of db+/db+ mice administered with saline (left) or green tea (right). Graph shows the peak intensity averages at m/z indicated, before (open column) and after (closed column) administration with saline (n = 4) or green tea (n = 4). The analyzed peak is indicated by arrows in the MS spectra. **P < 0.01; significantly different from the peak obtained before the administration, by unpaired *t*-test. Types of ProteinChip used were described in the Fig. 7 legend. Click here for file
