# Supplementary figures and images for: Effect of green tea on blood glucose levels and serum proteomic patterns in diabetic (db/db) mice and on glucose metabolism in healthy humans
Source: BMC Pharmacol. 2004 Aug 26;4:18. doi: 10.1186/1471-2210-4-18 (PMC517497; doi:10.1186/1471-2210-4-18)

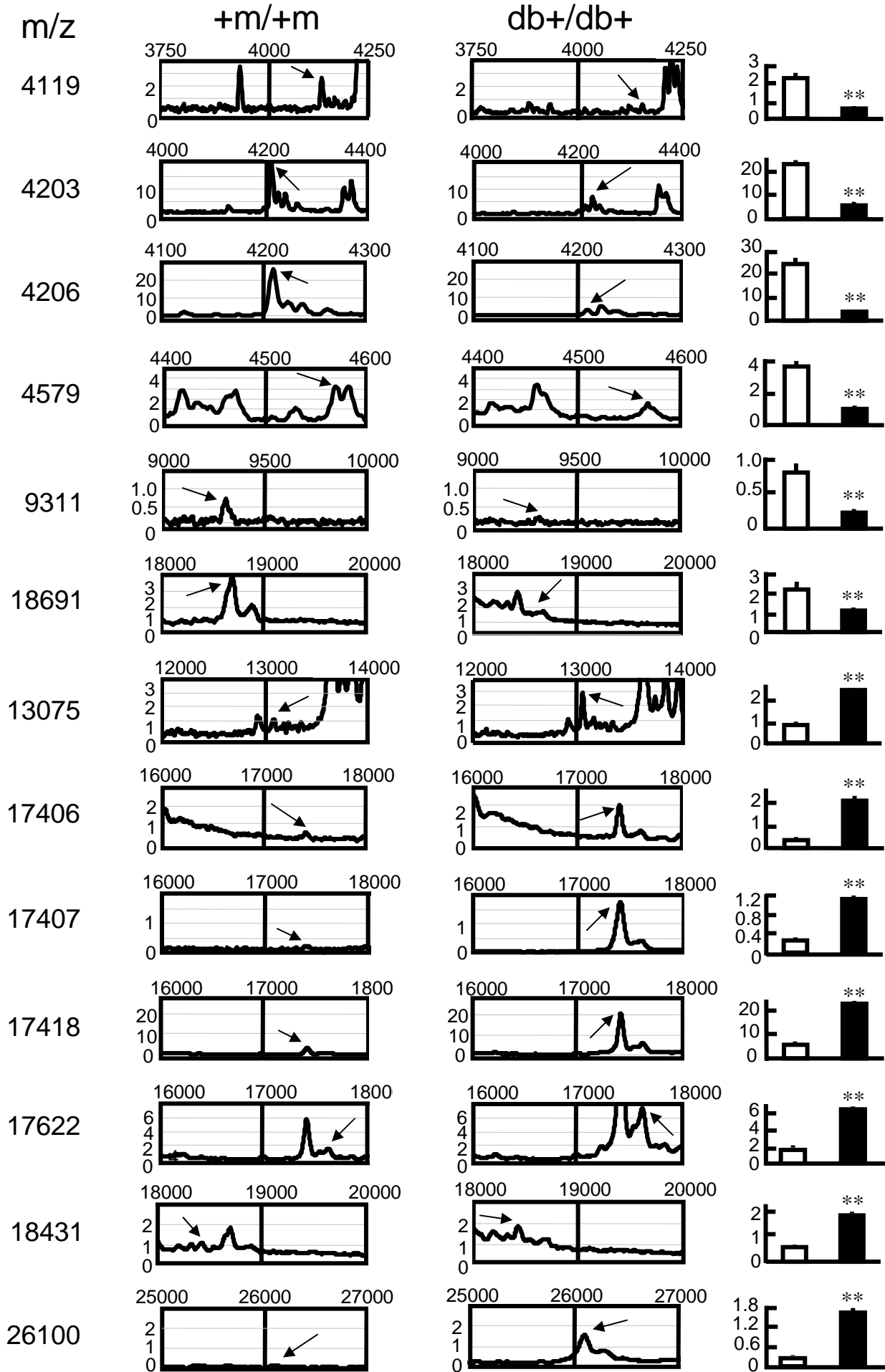

Supplement: Additional File 1 — Differences in serum protein profiles between diabetic and wild-type mice Typical data of relative peak intensities in +m/+m and db+/db+ mouse sera (left 2 panels, representative of 4–8 independent observations) and the peak intensity averages at m/z indicated (right panels; +m/+m: open column, n = 4; db+/db+: closed column, n = 8). The analyzed peak is indicated by arrows in the data of mass spectral signals. **P < 0.01; significantly different from the peak in wild-type mice, by unpaired t-test. Types of ProteinChip used were described in the Fig. 6 legend. [file 1471-2210-4-18-S1.pdf]

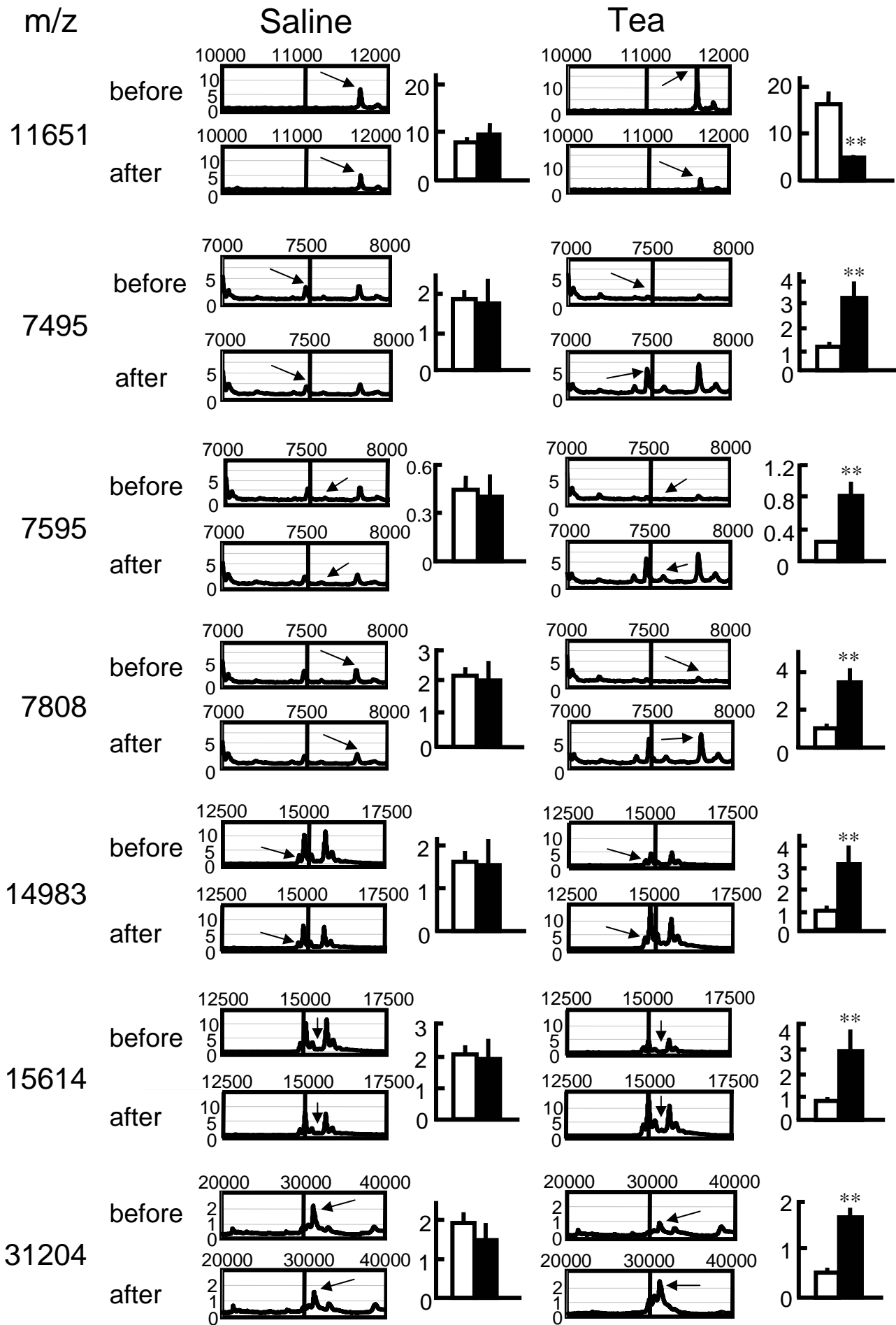

Supplement: Additional File 2 — Changes in serum protein profiles of db+/db+ mice after green tea administration MS spectra shows typical changes in the serum protein profiles of db+/db+ mice administered with saline (left) or green tea (right). Graph shows the peak intensity averages at m/z indicated, before (open column) and after (closed column) administration with saline (n = 4) or green tea (n = 4). The analyzed peak is indicated by arrows in the MS spectra. **P < 0.01; significantly different from the peak obtained before the administration, by unpaired t-test. Types of ProteinChip used were described in the Fig. 7 legend. [file 1471-2210-4-18-S2.pdf]
